# Perspective of Traditional Birth Attendants on Their Experiences and Roles in Maternal Health Care in Rural Areas of Northern Ghana

**DOI:** 10.1155/2018/2165627

**Published:** 2018-10-01

**Authors:** Peter Adatara, Agani Afaya, Elizabeth A. Baku, Solomon Mohammed Salia, Anthony Asempah

**Affiliations:** ^1^Department of Nursing, School of Nursing and Midwifery, University of Health and Allied Sciences, Ho, Ghana; ^2^Public Affairs Unit, University of Health and Allied Sciences, Ho, Ghana

## Abstract

**Background:**

Traditional birth attendants play significant roles in maternal health care in the rural communities in developing countries such as Ghana. Despite their important role in maternal health care, there is paucity of information from the perspective of traditional birth attendants regarding their role on maternal health care in rural areas in Ghana.

**Objective:**

The aim of this study was to explore and describe the role of traditional birth attendants in maternal health care in the rural areas in Ghana.

**Methods:**

A qualitative explorative approach was adopted to explore the role of traditional birth attendants in maternal health care in the rural areas of Ghana. Ten (10) out of a total of twenty-seven (27) practising traditional birth attendants in the study area were purposefully selected from five (5) rural communities in the Bongo District of Ghana for the study. Data were collected through in-depth, unstructured, individual interviews using a guide. Data collected from the interviews were transcribed verbatim and analysed to identify themes.

**Results:**

Six main roles of traditional birth attendants on maternal health care in rural areas were identified in this study: traditional birth attendants conduct deliveries at home, they provide health education to women on nutrition during pregnancy and lactation, they arrange means of transport and accompany women in labour to health facilities, they provide psychological support and counselling to women during pregnancy and childbirth, and traditional birth attendants are not paid in cash for the services they render to women in the rural areas.

**Conclusion:**

Our study brought to light the critical role traditional birth attendants play in maternity in rural and remote areas in Ghana. There is a need for skilled birth attendants to collaborate with traditional birth attendants in rural and deprived communities to provide quality and culturally accepted care in the rural communities.

## 1. Background

One of the strategies identified as being crucial for saving the lives of pregnant women and as an indicator for progress in the reduction of maternal mortality is the utilisation of skilled birth care [1]. Although skilled birth care is identified as the ultimate strategy for saving the lives of pregnant women and infants, in rural and deprived communities in developing countries such as Ghana, women continue to deliver at home with the assistance of traditional birth attendants (TBAs) [2].

The World Health Organisation (WHO) defines a traditional birth attendant as “a person who assists the mother during childbirth and initially acquired her skills by delivering babies herself or through apprenticeship to other traditional birth attendants” [3]. Studies show that, in the rural and deprived communities in sub-Saharan Africa, TBAs constitute the greater number of childbirth care providers due to the unavailability of skilled birth attendants such as midwives, nurses, and doctors [4]. Traditional birth attendants are known to have a long history as childbirth attendants in many communities in developing countries including Ghana, although TBAs are not considered skilled birth attendants by the definition of a skilled birth attendant [5].

The picture is not different in Ghana. TBAs still continue to play significant roles in maternal health care in the rural and deprived communities where there are inadequate skilled birth attendants. Studies have shown that in Ghana women have traditionally preferred to deliver at home because it is cheaper and easier as women who deliver at home receive social support from their extended families and do not have to pay much for the delivery services [6, 7]. According to reports from the Ghana Statistical Service (GSS), 62.9% of the deliveries in the Northern Region occurred at home assisted by nonskilled attendants, while in the Central and the Upper West Regions 39.1% and 38.6% births occurred at home assisted by TBAs and relatives, respectively [7]. The above statistics explained the significant roles TBAs play in the health of women and children in rural areas in Ghana. Studies established that one of the strategies to increase utilisation of skilled birth attendants where TBAs are the predominant providers of childbirth care particularly in the rural areas involves linking TBAs with the formal health care institutions and fostering collaborative practice with TBAs [8].

Despite the importance of TBAs in maternal health care in rural and deprived communities in Ghana, there is paucity of literature regarding the exact role of TBAs in maternal health care in rural communities where many women in labour patronise their services. The aim of this study was to explore the perspective of TBAs on their roles in maternal health care in the rural and deprived communities in Ghana. The findings of the study will also be useful to the Ministry of Health and Ghana Health Service and other stakeholders involved in formulating policies on facilitating the utilisation of childbirth care services in the rural areas of Ghana to reduce maternal mortality.

## 2. Design and Methods

### 2.1. Research Design

A qualitative explorative design was used to explore and examine the role of TBAs in maternal health care from their own perspectives in the rural communities in the Bongo District of Ghana.

### 2.2. Research Setting

The research was carried out in the Bongo District in the Upper East Region of Ghana. This district was selected as the setting for conducting the study because it is one of the most rural and deprived districts in Ghana and has all the characteristics of a typical rural area in Ghana [7]. Also, the Bongo District was chosen because it is one of the districts with low utilisation of skilled birth care provided by skilled birth attendants [9]. The Bongo District is amongst the ten (10) districts in the Upper East Region of Ghana, with Bongo Township as its capital. According to the Bongo District Health Directorate, there is only one doctor who is the Medical Superintendent of the district hospital. He manages the hospital and sixty-five nurses in the entire district [9]. There is only one district hospital with one general practitioner (medical doctor) and few midwives providing skilled birth care. The remaining maternity units in the health facilities are manned by midwives providing primary maternity care for women during pregnancy and childbirth.

### 2.3. Study Population and Sampling Method

The participants for this study included men and women who practised as TBAs in the rural areas in the Bongo District of Ghana. Participants were recruited using purposeful and snowball sampling strategies. Some of the TBAs who were identified by community opinion leaders were asked to invite other TBAs to participate in this study. A total of ten (10) TBAs out of twenty-seven (27) practising TBAs in the study area were recruited from five (5) rural communities from the Bongo District of Ghana. These TBAs were chosen for the study because they met the criteria and were willing to take part in the study. The researchers strived to recruit a maximum variation sample to include diverse experiences in the study. The researchers also selected and interviewed TBAs who have requisite experience on maternal health care in the study area.

The inclusion criteria included the following:

- Men and women who had practised childbirth care for more than a year.
- TBAs who live in the communities for more than one year.
- TBAs who were still in active service and were willing to participate in the study.

### 2.4. Data Collection and Procedure

Data were collected through semistructured individual interviews using a guide. The interview guide contained questions that inquired about the role of TBAs in maternal health care in the rural areas. Individual interviews provided a deeper understanding of social phenomena such as the role of TBAs in maternal health care that would be obtained from questionnaires [10]. Semistructured interviews were also appropriate for this study because the researchers required detailed insights from participants [10]. The in-depth interviews allowed the researchers the opportunity to define the areas to be explored and to probe further to pursue an idea or response in detail. The interviews were done face-to-face with participants at their various homes and at their convenient time which lasted between 45 to 60 minutes. The interviews were recorded with the permission of participants.

### 2.5. Data Analysis

Thematic analysis approach was used to analyse the data. We examined the qualitative data, which were categorized based on prominent theme patterns expressed in the text of the individual interviews [11]. We broke down the narrative data into smaller units, coding, and naming the units according to the content they represent and grouping coded material based on shared concepts and meanings. All transcripts were read and analysed by three members of the research team in a three-stage process of data analysis and synthesis [12]. In the first stage, we used the exploratory method of holistic coding for the first coding cycle. After reading all the transcripts, the three researchers met as a group to discuss patterns and units and to develop a coding protocol. The verbatim transcripts of the individual interviews were coded into different units. In the second stage of data analysis and synthesis, the focused coding method was used for the second coding cycle. The three researchers met together and, through peer discussion and agreement, recategorized the coding units. Finally, based on the coding, the primary researcher identified themes that integrated substantial sets of the coding units. Sets of categories were created to describe the predominant themes that emerged from the text [13].

### 2.6. Ethical Considerations

This study is part of a research thesis carried out toward the award of a doctorate degree. The research had ethical approval from the Ethical Committee of Faculty of Health Sciences Research Technology and Innovation Committee (FRTI) at Nelson Mandela University** (**H14-HEA-NUR-30**)**. The participants chosen for the interview were provided with both verbal and written information on the purpose, general content, and nature of the investigation. The participants were told that any information they provided would be kept confidential. Each participant was given time to read the letter. The consent forms were signed by each participant who could read and thumb printed by participants who could not read.

## 3. Results

Six (6) themes emerged from the data analysis with regard to the roles of TBAs in maternal health care:

- TBAs conduct deliveries at home in rural areas.
- TBAs provide health education to women on nutrition during pregnancy and lactation in our community.
- TBAs arrange means of transport and accompany women in labour to health facilities.
- TBAs provide natural family planning to women in rural areas.
- TBAs provide psychological support and counselling to women during pregnancy and childbirth.
- TBAs are not paid in cash for the services we render to women in the rural areas.

### 3.1. Profile of the Participants

The participants were made up of seven (7) women and three (3) men. All the participants had no formal education and could not read and write. Majority (8) of the participants were within the ages of 60 and 70. Only 2 of the participants were within the ages of 55 and 59. Majority of the participants have been practising childbirth care in their various communities for more than five years. All the participants apart from being childbirth care givers are also peasant farmers.

### 3.2. Roles of TBAs in Maternal Health Care in the Rural Communities

The TBAs during the individual interviews outlined four major roles they play in maternal healthcare in rural areas. The roles/themes are discussed below.

*Theme 1: TBAs Conduct Deliveries at Home in Rural Areas. *One of the major themes that emerged from this study is the role of TBAs in maternal health care in rural communities in Ghana and particularly in the Bongo District was that TBAs assist in conducting uncomplicated deliveries at home. The TBAs indicated that, due to the absence of skilled birth attendants in rural communities in the study area especially in the night and weekends, they are always called upon to assist women to deliver. These were captured in the following quotes:

“In the village here, we assist women to deliver at home especially in the night when they cannot go to the health facility to deliver”.

“I must say that I have been helping women to deliver in this village well over twenty years now. When the women are in labour and cannot go to the hospital or the clinic, they call on us and we have to assist them”.

“As for me I think, we have been doing the work of the nurses and doctors in this community because the trained nurses and doctors are not in this community and we are always around whether in the night or weekend we are there to attend to the women when in labour”.

The participants indicated that they always advise women to go to the health facility to deliver but sometimes they would refuse and insist that they prefer to be assisted by them.

“We normally advise the women to go to the health facility to deliver but they always refuse insisting that they would prefer their (TBA) services. And so, we must assist them to deliver”.

.... I have told many women in labour to go to the hospital where there are nurses and doctors to assist them to deliver but they would always refuse. Some would even tell us (TBA), they prefer to be assisted by us”.

Some of the participants reported that the labouring women usually prefer their services because the midwives and nurses are not in their community to conduct labour. If even at all, if women want to go to the health facility to deliver, they would have to travel more than 15 km to the nearest health facility to access skilled birth care. The participants indicated that it is more serious if the labour occurs in the night, where there is no health facility in the community or nearer. These have often compelled labouring women to rely on their services.

“I must say that we cannot blame the women for calling on us to assist them to deliver because there is no nurse or doctor in this community to provide the skilled birth care. And if the labouring woman wants to go to the health facility to deliver, they would have to walk more than 15 km to the nearest health facility. So, they would rely on us to help them instead of travelling that far”.

“.... We have been assisting the women to deliver because sometimes the labour occurs in the night and there is no nurse or doctor in this village to assist them to deliver. We have to help them because we are their doctors and nurses in this community”.

The participants indicated that even though they have been assisting them to deliver in their homes, any time they examine the woman in labour and feel that it is beyond their level, they advise the husband or relative to take her to the hospital to deliver. This was evident in the following quotes:

“I normally ask the husband to take the labouring woman to the hospital to deliver when I examine her and realise that it is beyond my level. We have been doing this work for many years and we are experienced even though we have not been to school to learn it”.

“I don't usually try to assist a woman in labour where I think based on my assessment of the woman during labour that it is beyond me. I will just be honest and tell the husband or any relative who is available to take her to the health facility for her to deliver”.

The participants reported that when they have challenges with women who are weak and pale or those delivering for the first time, they refer them to the health facilities to deliver.

“We have been doing this work for many years now. So, when you call on me and I see that you are delivering for the first time or the woman is looking weak or the eyes are looking whitish (pale), I know that this is not my case, I will not attempt. I will just ask the husband or whosoever is around to take her to the health facility to deliver”.

.........oh, I know them. I have done this work for a very long time. I am now even old, yet I still assist women to deliver. Because of my experience, when I am called and immediately I get there, I will assess the labouring woman and when I see that she has no blood or has ever delivered through operation, I will just ask her to go the hospital because I cannot give blood or operate to remove the baby”.

The participants indicated that although they have been doing this work for many years, they have never lost a woman through labour yet, they are not respected or regarded by the health personnel.

“I must say that since I started doing this work, I have never lost a woman or child through child birth. All the women I assisted to deliver are always happy and appreciative. Yet the nurses do not regard us.

“.... I assisted more than thirty (30) women to deliver since I started practising as a TBA and none of the women or the child died. But, we are not regarded or respected by the nurses. They think that we have not been to school”.

*Theme 2: TBAs Provide Health Education on Nutrition during Pregnancy and Lactation. *One other theme that emerged from this study regarding the role of TBAs in maternal health care in the rural areas in Ghana was that they provide health education on nutrition and natural family planning to women in their respective communities. The participants reported that although they have not been to school to learn about nutrition, their experience in childbirth care over the years has given them the opportunity to know the traditional foods that provide the right nutrition for pregnant women and nursing mothers.

“I must say that one of the things I do as a TBA in this community is to provide nutrition education to pregnant women and nursing mothers for healthy growth of the foetus or the baby. Many of the pregnant women especially the younger ones do not know what to eat and what not to eat during pregnancy. So, I mostly engage them with their mothers-in-law to educate them on what to eat and what they need to avoid when you are pregnant or nursing your baby”.

“Teaching mothers on the kind of food to eat during pregnancy and when nursing a baby has been part of my duties in this community. I have been doing this work for some time now and the women in this community know about it and would always seek my advice on nutrition when pregnant or after delivery”.

The participants also explained that even though they have not been to school they are much aware of the traditional food stuff that are available in their community that mothers do not have to spend lots of money to buy unlike nurses who usually advise and prescribe certain diets for women who cannot afford to buy them. These were captured in the following quotes:

“As I said earlier, we live in this community and we know the food stuff in this community that are good for pregnant women or the nursing mother. We usually do not advise or prescribe certain food stuff that are not available in this community unlike what the nurses have been doing. Most of the women in the community do not have money to go to market to buy all those things the nurses have been asking to do”.

“.... hmmm, as for me, I usually advice the pregnant or nursing mothers based on what they have in the family, so that they won't go out looking for money to buy. There are nutritious food stuffs in this community far better than what is sold in the market”

One of the participants of this study mentioned the food stuff in the community that are usually recommended or prescribed for pregnant and nursing mothers for the purposes of healthy growth of the foetus and the pregnant mother herself, thereby enhancing breast milk production in the case of nursing mothers and boosting the immunity of the mother.

“The food stuff that I usually recommend for pregnant mothers which are available in this community and everyone can afford them include ‘Kolgo' in Frafra, also known as dawadawa (locust bean boiled and fermented), smoked guinea fowl, beans, dry okro soup and bitor soup (local vegetable soup) and bitter-leaf soup. All the above-mentioned foods are equally good for nursing mothers, but we usually add ‘zoomkom and pusakoom'. Zoomkom is local drink prepared from millet flour and fruit, tooro (baobab tree fruits) to provide energy and facilitate breast milk production for the nursing mothers”.

Some of the participants indicated that there are certain foods that are to be avoided because they are not good for the mother and the child.

“We usually recommend that pregnant women should not be eating too much pepper, certain types of fish such as swordfish and king mackerel. I realised throughout the years of my practice that any time pregnant women eat these types of foods they encounter problems. So, we advise them to avoid them”.

Majority of the participants reported that although there are taboos in the community prohibiting pregnant women from eating certain foods, there are always alternatives available.

“I must acknowledge that in this community, women especially pregnant women are prohibited from eating chicken, snails, dog meat, cat meat and monkey meat. But, we can always find alternatives to these foods”.

*Theme 3: TBAs Arrange Means of Transport and Accompany Women in Labour to Health Facilities. *This study revealed that TBAs arrange means of transport and accompany women in labour to health facilities to give birth. The participants indicated during the interviews that there were occasions where they (TBAs) had to arrange transport for labouring women and accompanied them to health facilities to give birth.

“I must indicate that there were times, I personally had to arrange for a taxi cab or motor cycle to transport women in labour to a health facility to give birth. In many of such occasions, I had to personally accompany the labouring woman to the health facility to give birth because none of the relatives was around to do that”

Another participant also indicated how she accompanied a labouring woman to a health facility and had to pay for a drug that was prescribed but was not in the health facility.

“We do a lot of things for women especially during labour in this community. There was a day I had to accompany a labouring woman to give birth in the hospital. When we got to the hospital, the doctor prescribed a drug for her to buy but she didn't have money. I had to use my own money to buy the drug for her”

The participants reported that, because of the long distance that women had to travel to health facilities in the study area to give birth, relatives of labouring women usually prefer TBAs to accompany labouring women in case they deliver on the way to the health facility.

“The reason why we (TBAs) accompany labouring women to health facilities to give birth is because of the long distance that labouring woman would have to travel to the health facility to give birth. So, we go with them to the health facility especially if the means of transport is a taxi cab and in case the baby is coming out on the way to the health facility, we can assist her to give birth”.

Moreover, participants reported that, apart from the relatives asking them (TBAs) to accompany labouring women to health facilities to give birth, midwives working in the health facilities usually plead with them to always accompany labouring women to health facilities to give birth especially if it was in the night.

“We were advised by the midwives in the health facilities to accompany labouring women to the health facilities to deliver especially if it is in the night”.

“I was personally told by the midwives in the health facilities that I should always support and accompany labouring women to health facilities to give birth. So, because of the advice from the midwives, I had to always arrange transport and accompany labouring women to the health facility to give birth”.

It was also revealed in this study during interviews with the study participants that TBAs were motivated by midwives in the health facilities in the study area to always accompany labouring women to give birth in the health facilities. TBAs are usually given a bar of soap and sometimes some money by midwives to motivate them to accompany women to health facilities to deliver.

“The midwives in the health facilities motivate us to accompany labouring women to the health facilities to give birth. Anytime I accompany a labouring woman to a health facility to give birth, the midwives give me a bar of key soap to wash my clothes and sometimes, they give me some money to buy whatever I want to buy”.

*Theme 4: TBAs Offer Education to Couples on Natural Family Planning Methods. *Apart from education on nutrition during pregnancy and lactation, TBAs offer education to couples on natural family planning methods.

“One thing I also do as part of my work in this community is to educate young couples how to space their childbirths. I have done this over the years in this village and couples especially women patronize my services a lot and it is helping them”.

“We also educate married women on how to study their menstrual cycle to avoid pregnancy if the child is not yet three years or more. This has helped many of them to avoid giving birth every year to create problems for themselves and their children”.

Some of the participants indicated that the couples patronise their education on family planning because it has no side effects and they do not pay any money for it as compared to what the nurses offer to them in the health facilities.

“.... what I have heard from the women who patronize our services on family planning is that they don't experience any problem using the natural family planning unlike the drugs and injections the nurses give them. Apart from that they don't pay any money for our services”.

The women like our services because they have nothing to lose. They won't pay money for it and they will not experience any problem after they have used the natural family planning method”.

Although participants indicated that the natural family planning methods have no side effects, it is sometimes difficult for some couples to strictly follow the instructions. Some women miscalculate their menstrual cycle and end up becoming pregnant.

“I can't say that we have been achieving 100% results in our education on natural family planning methods as a way of spacing childbirth. There are times we give instructions to women and they end up not following it. And when that happens, they end up becoming pregnant when the child is less than one or two years”.

“I must say that some women fail to follow instructions and they become pregnant unaware. But when they become pregnant as married couples and the baby is still less than two years, we counsel them to keep the pregnancy and take good care of the child. Our tradition does not permit a woman especially a married woman to abort pregnancy”.

*Theme 5: TBAs Provide Psychological Support and Counselling to Women during Pregnancy and Childbirth. *The interviews with the participants revealed that one of the roles that the TBAs play in maternal healthcare in rural and deprived communities is the provision of psychological support and counselling to women during pregnancy and childbirth. The participants said the provision of psychological support to women during pregnancy and childbirth is what makes their childbirth services patronised by women in their communities.

“We usually provide psychological support and counselling to women during pregnancy and childbirth. Labour is a painful thing and therefore, women going through the pain need support and advice to go through the delivery process with confidence”.

“We sometimes provide psychological support to labouring women to have confidence to tolerate the pain especially the young mothers who are delivering for the first time that they can make it.

Participants indicated that the psychological supports they provide to labouring women and pregnant women range from massaging their back to praising them for their efforts during labour. They explained that the psychological support is supposed to encourage women to put up their best in the labour process and to have a positive attitude toward the delivery process.

“We massage them and keep patting their back and encourage them that they can make it with little effort. We encourage them to have a positive attitude toward the labour”.

“One of the things I usually do to women when they are in labour is to keep on praising them in whatever they do and inform them they need to do more for the baby to come out early so that they can rest. Sometimes, I have to join other women to sing for the labouring woman to help her minimize the labour pain and to encourage them to push harder for the baby to come out”.

The participants explained that providing psychological support to women during labour is one of the major roles that makes them enjoy their services. The participants indicated providing psychological support is what is lacking in the health facilities that make some of the women in the rural areas not patronising the birth care services of skilled birth attendants in rural areas.

“I must say that things like singing for women during labour, praising them, massaging their back, encouraging them during labour and not shouting at them is what makes our services different from what is done in the health facilities. These things are not done in the health facilities. And it is the more reason why some women do not like going to deliver in the health facility”.

“............Psychological support is very important in labour management. We usually do that to motivate the labouring women to deliver without so much stress. One reason why most of the women do not like going to health facilities to deliver but keep on calling us anytime they are in labour is because of the support we provide them”.

Some of the participants indicated that labour is a natural life event that birth care providers need to understand and encourage labouring women to go through it successfully without any problem. They recommended that skilled birth attendants should cultivate the habit of supporting labouring women psychologically and allowing relatives to assist them when they are busy.

“I know nurses and doctors are always busy and are not able to provide the needed psychological support to labouring women. But, what they need to understand is the fact that labour is a natural life event and therefore, labouring women should be encouraged to go through it successfully without any problem or complaint”.

“It is bad to hear that in the health facilities, nurses shout at women who are in labour. Labour is a natural life event and sometimes the labouring women do not know what they are doing and so, they need encouragement and not insults and shouting at them. If the nurses are always busy they can allow relatives to assist them provide the support the labouring woman needs”.

Some of the participants indicated that there were times they accompanied labouring women to a health facility to be attended by the healthcare professionals and instead of appreciating and encouraging them to do more, they rather blame them for detaining the woman for a long time before bringing her to the health facility.

“I felt very bad when I had to accompany a woman who was in labour and came to me to take her to a health facility. Upon getting to the health facility, the midwife after receiving the woman, did not even examine her to know the stage of the labour process or the progress of the labour but just started blaming me for keeping the woman for a long time before bringing her. She would not listen to me for any explanation. And I really felt bad that day and since then I did not accompany any labouring woman to a health facility again”.

“I must say that the nurses do not want to appreciate what we the TBAs do to help labouring women in the community. Instead of they (the midwives) allowing us to work together to see how we can help these women they are rather blaming us for causing problems for them. The midwives think that because we haven't been to school, so we don't know anything about assisting women to deliver. But, you are not available in this community to help them when they are in labour and need you. Is half a loaf of bread not better than nothing”?

*Theme 6: TBAs Are Not Paid in Cash for the Services We Render to Women in the Rural Areas of Ghana. *The TBAs indicated that although they have been assisting women in maternal health related care in the rural areas of Ghana, they are not paid in cash for the services they render to the women in the rural communities in the study area. Although TBAs are usually paid in kind by the husband or relatives in the forms gifts such as soap, guinea fowls, fowls, and goats, they are not paid any allowance by government or anybody for the care they render to women in rural areas. Their services are on sacrificial basis and not for a fee. This practice has often left them struggling to take care of their own needs although what they do put so much stress on them.

“....... As for our work, it is not a paid work, I do it for free and I have never charged any woman before assisting her to deliver. So, we don't benefit anything from what we do in terms of money but just the joy you get when you are able to see a woman deliver successfully.”

“......I must say that we don't benefit anything from this work we are doing. We are not paid any money for rendering any service to women in this village. It's our challenge because we do this work and sometimes, the women are so poor that majority of them are not able to afford to buy soap for us to wash our clothes”.

Participants mentioned that although they render the services in maternal health care in the rural areas that should have been done by trained skilled birth attendants such as midwives and doctors who are paid by the government, they are not given anything by the health sector neither are they recognised.

“We have been doing the work of the nurses who are paid monthly to do that and yet the government does not give us anything. They don't even recognise that we are doing their work for them”.

“I must say that some of the nurses who come to this community to work do not have anything to do because we do their work for them and yet we are not paid a penny”.

Participants suggested that there is the need for the government to support them with logistics and some allowances to motivate them to put up their best in helping and supporting the poor women in the rural communities who cannot afford to travel long distances to access skilled birth care. These suggestions were captured in the following quotes:

“.... I think government should support us by giving small money and supporting with things such as gloves, detergents and aprons to enable us do our best to assist these poor women who usually rely on us for assistance”.

“I do not understand why, the government and the health care managers in Bongo would not support us. We are doing all this work for them at least they should give us something small if not every month may be every three months to motivate us to put up our best”.

## 4. Discussion

Our study sought to explore and describe the role of traditional birth attendants in maternal health care in the rural areas in Ghana. TBAs have long been practising as traditional midwives in rural areas of Ghana and yet, there is a lack of recent empirical evidence on the roles TBAs play in maternal health care in rural areas of Ghana. The present study found that one of the roles TBAs play in maternal health care in the rural communities in Ghana is that they conduct deliveries at home. The participants indicated that although they have not been formally trained to conduct deliveries, they have been assisting women deliver at home in their communities over the years. The results depict that TBAs in the rural communities of Ghana are highly utilised in childbirth care because there are no skilled birth attendants in the rural areas especially during the nights and weekends to attend to women who are in labour. It should be noted that, in the absence of a skilled birth attendant in the community, labouring women would have no options than to patronise the services of TBAs. After all, half a loaf of bread is better than nothing. The present finding is consistent with findings from recent studies, which have shown that TBAs continue to assist women in labour to deliver in some rural communities in developing countries [14, 15], for instance, in a qualitative study to assess stakeholder views/on the incorporation of TBAs into the formal health systems of low- and middle-income countries. It was concluded that TBAs are involved in the provision of pregnancy care [16]. These findings imply that healthcare managers in developing countries need to develop strategies of training TBAs and facilitating a collaborative practice amongst TBAs and midwives in rural communities as a short-term solution till such a time where there are adequate midwives in the rural communities to reduce maternal mortality. This study findings point to the fact that, in rural communities in developing countries such as Ghana, although the strategy of encouraging the utilisation of skilled birth attendants during childbirth is a laudable idea, it is difficult to achieve in a situation where the skilled birth attendants are not available in the community and TBAs are the only care providers available. There is the need for skilled birth attendants to partner with TBAs in rural areas to provide woman-centred childbirth care.

Moreover, the present findings suggest that, apart from the traditional role of assisting women to deliver in rural communities, TBAs equally perform critical and important functions such as providing education on nutrition during pregnancy and lactation. These roles are often performed by TBAs taking into consideration the cultural values and tradition of the women and the community at large, thereby wining the confidence of the labouring women and their family. Recent studies have highlighted that because TBA-client relationships are often positive and incorporate shared values and belief, the flexible attitudes of TBAs regarding payment are the factors that usually influence many home births in rural communities in developing countries [8, 16, 17]. What is interesting about the present findings is the fact that TBAs provide education on nutrition during pregnancy and lactation based on the available local nutritious foods that would meet the nutrition needs of the pregnant women or the lactating mothers and could be afforded by most families. This is an important finding that could be adopted and be practised by midwives when educating pregnant women and lactating mothers during antenatal and child welfare clinics to ensure women practise it. Healthcare managers of the institutions in developing countries need to know how these practices could be integrated into the skilled birth care organisation in ways that would ensure women's needs are better met.

It was also revealed in this study that one of the maternal health care roles of TBAs was to arrange means of transport and accompany labouring women to health facilities to give birth. It was reported in previous studies that TBAs play important role in improving maternal health care in rural areas by referring women in labour and at times, accompanying them to health facilities for childbirth [18, 19]. Companionship during child birth by TBAs offers physical and social support to labouring women as they are always anxious about the unknown outcome of the childbirth. The finding of this study implies that TBAs could be used as referral agents in rural communities to promote skilled birth care and provide social and psychological support. Also, it was discovered in this study that TBAs provide counselling and education on natural family planning to women in rural communities in the study area. It was revealed that although some of the women in this study knew about modern family planning contraceptives, some of them still seek education on natural family methods such as the calendar method and abstinence from sexual intercourse as a form family planning from TBAs. This finding is consistent with previous studies that indicated that TBAs offer services such as traditional methods of family planning to couples who do not want to use modern contraceptives in rural areas [14]. This implies that TBAs could also be used to educate women on the use of modern family planning methods to prevent unwanted pregnancies and sexual transmitted infections in the rural areas.

Providing psychological support and counselling to women during pregnancy and childbirth was found in this study to be other important role TBAs play in maternal health care in the study area. Previous studies reported psychological support and counselling to women during pregnancy and childbirth as one of the reasons accounting for home births in developing countries such as Ghana [20, 21]. For instance, in a qualitative study conducted to identify the reasons that accounted for home births and use of traditional birth attendants in rural Zambia, it was found that most women utilised the birth services provided by TBAs because they perceived them to be respectful, skilled, friendly, and trustworthy and they provide psychological support to women during childbirth [21].

Although TBAs play important roles regarding maternal health care in the rural communities of Ghana, it came to light in our study that they are not paid in cash for the services they render to women in the rural areas of Ghana. Although, TBAs are usually paid in kind by the husbands or relatives in the form of gifts such as soap, guinea fowls, fowls, and goats, TBAs are not supported with logistics and allowances to motivate them to hold the fort in rural and hard to reach communities till such time that midwives are posted to those communities. It is important for healthcare managers and policy planners to identify all available childbirth care providers in rural communities and support them in terms of logistics and some allowances as a form of motivation to enable them to perform their roles. Skilled birth attendants should foster collaboration with TBAs in the rural communities to ensure that some refresher training is given to TBAs with regular monitoring and supportive supervision to ensure that they work within their limits and those that are beyond their level are quickly referred to health facilities for expert management. The training of TBAs is found in literature to be an effective way of enhancing their roles in rural areas [22].

### 4.1. Limitation of the Study

Although this study had some unique findings, it also had some limitations. This qualitative research about TBA focused particularly on their viewpoints and the roles they play in maternal health care in rural areas in Ghana. Including the viewpoints of other stakeholders such as skilled birth attendants on the roles of TBAs in rural communities would have enriched the findings. Also, the interviews were conducted with 10 participants which are not adequate to generalise the findings of this study.

## 5. Conclusion

It was concluded from this study that TBAs play critical role in maternal health in rural and remote areas in Ghana. This study identified the roles of TBAs in maternal health care in rural areas to include assisting women during delivery, providing health education on nutrition during pregnancy and lactation, providing natural family planning to women and providing psychological support, and counselling to women during pregnancy and childbirth. There is need for skilled birth attendants to collaborate with traditional birth attendants in rural and deprived communities to provide culturally accepted care in health facilities to facilitate the utilisation of birth care service.

## 6. Implication for Nursing Practice

The findings of this study have implications for nursing practice. The findings of this study call for collaborative practice between skilled birth attendants and traditional birth attendants to ensure that TBAs are supported in terms of training needs, logistics and motivation in the form of allowances to put up their best in maternal health care in rural and hard to reach communities where there are no or few skilled birth attendants. There is also the need for health care providers and policy makers to develop strategies of training TBAs especially on complications of labour and referral to health facility for expert's management.

## Data Availability

The data used to support the findings of this study are available from the corresponding author upon request.
